# Suppression of *Pepper mild mottle virus* (PMMoV) by Modified Whey Proteins

**DOI:** 10.3390/life12081165

**Published:** 2022-07-30

**Authors:** Mohsen Mohamed Elsharkawy, Abdulaziz A. Al-Askar, Ahmed Abdelkhalek, Said I. Behiry, Muhammad Kamran, Mostafa Ali

**Affiliations:** 1 Department of Agricultural Botany, Faculty of Agriculture, Kafrelsheikh University, Kafr El-Sheikh 33516, Egypt; 2 Department of Botany and Microbiology, College of Science, King Saud University, P.O. Box 2455, Riyadh 11451, Saudi Arabia; aalaskara@ksu.edu.sa; 3 Plant Protection and Biomolecular Diagnosis Department, ALCRI, City of Scientific Research and Technological Applications, New Borg El Arab City, Alexandria 21934, Egypt; aabdelkhalek@srtacity.sci.eg; 4 Agricultural Botany Department, Faculty of Agriculture (Saba Basha), Alexandria University, Alexandria 21531, Egypt; said.behiry@alexu.edu.eg; 5 School of Agriculture, Food and Wine, The University of Adelaide, Adelaide, SA 5005, Australia; muhammad.kamran@adelaide.edu.au; 6 Department of Food Technology, Faculty of Agriculture, Kafrelsheikh University, Kafr El-Sheikh 33516, Egypt; mostafa.ali@agr.kfs.edu.eg

**Keywords:** *Pepper mild mottle virus*, pepper, quercetin, whey protein, UHPLC-ESI-Q-TOF-MS, systemic resistance, RT-PCR

## Abstract

Modified whey proteins with quercetin (WPI-QU) and onion extract (WPI-OE), as a control approach, could be applicable because it is available, safe and cheap. The modified whey protein isolate (WPI) with quercetin dihydrate and onion extract powder rich with quercetin were evaluated for induction of systemic resistance against *Pepper mild mottle virus* (PMMoV) in pepper plants. Data of mass spectrometry illustrated that one or more of Qu isomers covalently attached to WPI. Unmodified whey protein (UWPI), WPI-QU and WPI-OE significantly decreased PMMoV concentration and severity at two weeks after inoculation. Plant height, number of leaves, and shoot fresh and dry weights were substantially increased in WPI-QU- and WPI-OE-treated pepper plants compared to the control. Total antioxidant status (TAS) and vitamin C contents were highly increased in WPI-OE-treated plants compared with other treatments. The expression levels of defense related genes (*PR4*, *PR9*, *TIN1* and *PIN2*) were enormously elevated in WPI-OE and WPI-QU treatments using qRT-PCR. In conclusion, the results give novel insights to possible applications of the WPI–quercetin bioconjugates in designing a wide range of functional products. Moreover, this study is the first to establish the effective control of PMMoV by modified whey proteins.

## 1. Introduction

Whey proteins have been considered a waste product that is produced during milk and cheese processing. On the other hand, polyphenols such as flavonoids, which contain two phenyl rings and a heterocyclic ring, are considered the secondary metabolites group [1]. Many fruits, berries and grains contain quercetin, a bioactive flavonoid. Onion is a rich source of dietary quercetin found in aglycone forms such as quercetin 4-*O*-*β*-glucoside and quercetin aglycone. It is widely studied because of its pharmacological properties such as anti-cancer, antioxidant and antimicrobial activity [2]. In the presence of oxygen, quercetin may be oxidized to a quinone radical, which can then combine with nucleophilic amino acids in proteins, such as lysine or tryptophan, to produce protein–quercetin bioconjugates [3]. Proteins covalently modified with phenolic compounds could act as antioxidants and antimicrobials [4]. 

Sweet and hot peppers are essential vegetables all over the world. Sweet and hot peppers can be cultivated both in greenhouses and the field. Pests and diseases are severely destructive to pepper production. Plant disease losses may have a major economic effect, resulting in lower income for pepper growers and distributors, as well as higher costs for consumers. Plant viruses are considered the main limiting factors for pepper production [5]. Among viral pathogens affecting pepper production, *Pepper mild mottle virus* (PMMoV) is one of the most harmful viruses for pepper fruits [6]. The symptoms of PMMoV include dwarfing, mottling and deformed leaves and fruits [7]. Just before harvesting, symptoms appear, causing severe losses in crop production [8]. The severity of infection is varied depending on the plant age and the cultivar; however, in general, the yield losses can reach 97% [7]. PMMoV is very stable, and has been found in plant debris, greenhouse structures, pots and horticultural equipment over long periods. Although no insect vectors have been identified, humans may be considered the pathogen’s primary vector [9]. In order to manage the PMMoV disease, farmers must follow proper sanitation practices and sow only clean seeds. As a *Tobamovirus* member, the only way to control PMMoV is to avoid it. Milk has been shown to reduce *Tobacco mosaic virus* (TMV) infection in pepper, tomato and tobacco plants. Whey is a diverse and rich mixture of associated proteins with a broad range of biological properties. Modified and unmodified whey proteins were effectively used against viral pathogens in humans [10,11]. 

There is currently a lack of evidence regarding the potential of modified whey protein with phenolic compounds on plant viruses. Therefore, the main objectives of this study were to use fluorescence quenching, RP-HPLC and UHPLC-ESI-Q-TOF mass spectrometry to characterize the covalent interactions between whey protein and quercetin dihydrate (Qu) and onion extract powder rich with quercetin (OE), and to investigate the effect of covalent modification of whey protein with Qu and OE on induced resistance against PMMoV as a novel and safe strategy to control the virus. 

## 2. Materials and Methods

Quercetin dihydrate (Qu), was obtained from Alfa Aesar (Karlsruhe, Germany) and onion extract (OE, *Allium cepa* L., contained 95.49 ± 1.5% quercetin on a dry weight basis) was purchased from Rudolf Wild GmbH & Company KG (Eppelheim, Germany). Trolox (6-Hydroxy-2,5,7,8-tetramethylchroman-2-carbon acid) and 2,2′-azinobis (3-ethylbenzothiazoline-6-sulphonic acid, ABTS) were bought from Sigma-Aldrich, Seelze, Germany. The whey protein isolate (WPI, 97.7% protein) was purchased from BiPRO, Davisco Foods International, Inc., Eden Prairie, MI, USA. All used chemicals were of analytical grade.

### 2.1. Modification of Protein with Quercetin Dihydrate and Onion Extract

Modification of WPI with Qu and OE was done as outlined in Rohn et al. [3] with minor modifications. WPI (1 g) was solved in 45 mL of distilled water, and the pH was adjusted to 9 using NaOH solution; then, the mixture was stirred gently for 1 h until complete hydration. Thereafter, 60 mg of each OE and Qu was added to ethanol solution (5 mL), and pH was readjusted again to 9. The interactions were done overnight at 25 °C with continuous stirring in the presence of air. The solutions were dialyzed at 25 °C for 24 h against water, followed by freeze-drying at −20 °C.

### 2.2. Determination of the Change in the Structure of Modified Proteins

#### 2.2.1. The Change in Tryptophan Fluorescence Intensity and the Hydrophilic and Hydrophobic Character of Proteins

The difference in tryptophan fluorescence intensities of unmodified and modified WPI proteins was determined [12]. RP-HPLC method was used to study the change in hydrophilic and hydrophobic character. The wavelength was detected at 205 nm.

#### 2.2.2. UV-Vis Spectrometry Measurements

The protein solutions were prepared in 5 mM PB buffer (pH 7.2), then filled in a quartz cell and scanned using a wavelength range of 190–600 nm. The scan rate was 300 nm/min and the data interval was 1 nm.

### 2.3. Determination of Chemical Changes of Modified Proteins Using UHPLC-ESI-Q-TOF Mass Spectrometer Analysis

Protein (1 mg) was dissolved in acetonitrile solution (1 mL of 1:1 acetonitrile: ultra-water on 0.1% formic acid). A Dionex–UltiMate 3000 UHPLC System with an EC 125/2 NUCLEODUR C8 Gravity Column was used to conduct the chromatographic analysis, a 5 µm column at 30 °C with a flow rate of 0.250 mL/min. Water (MS-Grade) with 0.1% formic acid (solvent A) and acetonitrile (MS-Grade) with 0.1% formic acid (solvent B) were used as the solvents. A syringe pump was used to inject samples directly into the instrument.

An ESI-Q-TOF mass spectrometer (micrOTOF-QII, Bruker Daltonics, Bremen, Germany) was used to examine the protein–phenolic bioconjugates. A capillary heated to 180 °C was used in the positive Ion-Mode for direct injection and 210 °C for LC-MS. Full scan data acquisition was carried out (50–3000 *m*/*z*). The Compass Data Analysis 4.2 program (Bruker Daltonics, Bremen, Germany) was used to process the precise mass data of the molecular ions.

### 2.4. Effect of Modified Whey Proteins on Pepper mild mottle virus (PMMoV)

#### 2.4.1. Plants and Pathogen

Pepper (*C. annuum* cv. Charlee) seedlings were obtained from the Agriculture Research Center, Kafr Elsheikh, Egypt. Seedlings were transferred to pots (20 cm in diameter) filled with sand and peat mixture (1:1). Pots were kept in the greenhouse at 24–26 °C for a 15 h photoperiod. The virus was isolated from naturally infected pepper plants showing symptoms suggested to be due to PMMoV. The virus was isolated from a single local lesion obtained from an infected *Nicotiana glutinosa* plant. PMMoV was propagated on *Nicotiana benthamiana* and *C. annuum* plants. Virus inoculum was prepared by grinding infected leaves in a 0.25 M potassium phosphate buffer (1:10; pH 7.1) using a sterilized and cooled mortar and pestle, followed by centrifugation at 12,000× *g* for 4 min. The virus was inoculated using the supernatant onto carborundum-dusted pepper leaves [13]. The plants were kept for at least 2 weeks for visual examination of symptoms and serology confirmation by ELISA.

#### 2.4.2. Effect of Modified Protein Treatments on PMMoV Disease Incidence and Severity in Pepper

Two week old pepper seedlings were sprayed with each of WP, WP-Qu and WP-OE proteins (1 mg mL^−1^). Plants treated with distilled water (DW) were used as a control. Seedlings were mechanically inoculated with PMMoV on the bottom leaves at 48 h after protein treatments. The severity of PMMoV was graded visually on a 1- to 5-point scale (no disease symptoms (1), mottling and mild symptoms (2), reduced leaf size and mosaic (3), stunting and severe mosaic (4), and very severe mosaic, stunting and malformation (5)). The experiments were carried out three times, with each treatment consisting of 12 plants. Out of 12 randomly chosen plants, the percentage of plants showing viral symptoms was calculated. Pepper growth parameters (plant height, fresh and dry weights and leaf number) were estimated at 5 weeks after virus inoculation.

#### 2.4.3. Effect of Modified Whey Proteins on PMMoV Concentration in Pepper Plants

PMMoV accumulation was measured by indirect ELISA to confirm the existence of virus [14]. Kits of ELISA were purchased from SANOEL, Santee Animal, Paris, France. Pepper leaves were collected at two and three weeks after virus inoculation. Each sample was ground in phosphate-buffered saline with Tween 20, and was put individually on ELISA 96-well plates, and kits were used. Absorbance values (A 405 nm) were measured on a microplate reader (Bio-Rad, Hercules, CA, USA).

#### 2.4.4. Quantitative RT-PCR (qRT-PCR)

Total RNA was extracted from treated and control pepper leaf tissues at 1 day after modified protein treatments (just before PMMoV inoculation) and 1 day after virus inoculation using Tri reagent (Molecular Research Inc., Cincinnati, OH, USA). cDNA synthesis protocol was performed [15]. The RT-PCR primers used are shown in Table 1. The 7500 real-time PCR system was used to conduct qRT-PCR. The data were analyzed using the ABI PRISM 7500 Software Tool from Applied Biosystems. The relative RNA quantification was measured by the comparative 2-CT technique [16].

#### 2.4.5. Assessment of Defense Enzymes and Total Phenols

Pepper leaves (1 g) collected at 2 weeks after inoculation (WAI) from treated or control plants were grounded with sodium acetate buffer (pH 5.5, 0.1 M) in a sterilized mortar and pestle. The tissue powders were stressed through a cheese cloth followed by centrifugation at 8000 rpm for 10 min, and the supernatant was utilized as a source for enzymes and total phenol evaluation. Peroxidase and polyphenol oxidase were measured [17]. Total phenol contents were quantified following the method of Folin–Ciocalteu [18].

#### 2.4.6. Assessments of Total Antioxidant Status (TAS) and Vitamin C

Total antioxidant status (TAS) was measured using the protocol described by Erel [19,20]. The oxidative effect was determined spectrophotometrically at 444 nm for 3 min. Vitamin C in pepper fruits was assessed by measuring the reduction in ascorbate concentration following the FRASC assay [21].

### 2.5. Statistical Analysis

A completely randomized design was used for the experiments. Experiments have been replicated at least thrice. Analysis of variance was performed using EKUSERU-TOUKEI 2010 (SSRI, Tokyo, Japan). A Fisher’s least significant difference was used to separate the means test at *p* ≤ 0.05.

## 3. Results

### 3.1. Characterization of the Change in the Structure of Modified Proteins

#### 3.1.1. The Change in Tryptophan Fluorescence Scans and Hydrophilic/Hydrophobic Character of Modified Proteins

**Figure 1:**
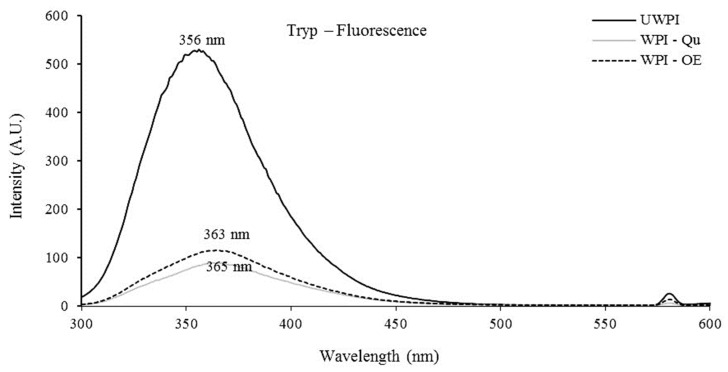
Tryptophan fluorescence scans of whey protein isolates modified with quercetin and onion extract. UWPI, unmodified protein; WPI-Qu, modified WPI with quercetin; WPI-OE, modified WPI with onion extract.

Phenolics were shown to be attached to proteins by observing the tryptophan fluorescence intensity change, and the findings are presented in Figure 1. The results revealed that the unmodified WPI displayed the maximum fluorescence intensity at 356 nm. Modified WPI proteins, on the other hand, exhibited a slight change in maximum emission wavelength, with measurements of 363 and 365 nm for WPI modified with OE and Qu, respectively. These findings revealed that Qu and WPI protein interaction was more significant than OE.

**Figure 2:**
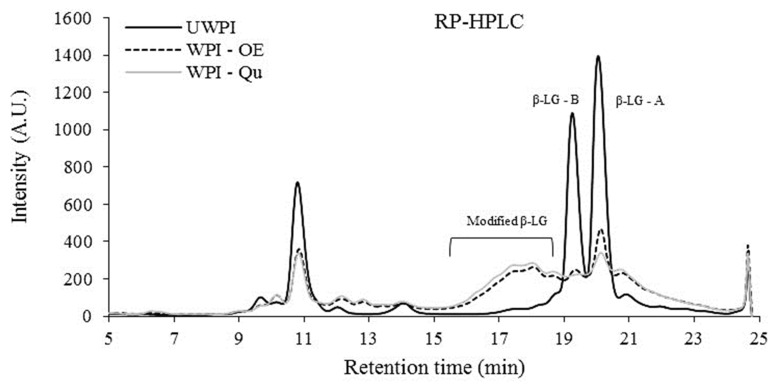
RP-HPLC of whey protein isolates modified with quercetin and onion extract. UWPI, unmodified protein; WPI-Qu, modified WPI with quercetin; WPI-OE, modified WPI with onion extract.

Changes in the hydrophilic/hydrophobic nature of the WPI-OE and WPI-Qu compared to unmodified WPI protein may be explained by changes in the major peak retention durations using RP-HPLC (Figure 2). The intensity of the major peaks (α-LA and β-LG) of modified WPI was reduced compared to unmodified WPI, as seen in Figure 2. The shift in hydrophilic/hydrophobic character indicates the unfolding of the modified proteins.

#### 3.1.2. The Change in UV-Vis Spectra of Proteins

**Figure 3:**
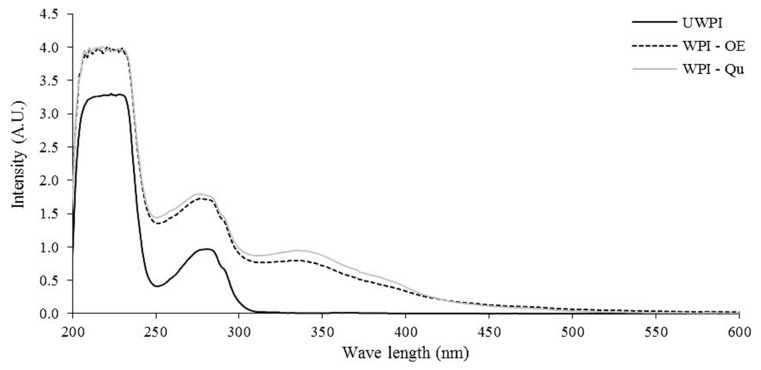
UV/Vis spectra of whey protein isolates modified with quercetin and onion extract. UWPI, unmodified protein; WPI-Qu, modified WPI with quercetin; WPI-OE, modified WPI with onion extract.

The results showed that the maximum of Qu absorption was recorded at ~350 nm. The unmodified WPI recorded only one peak with an absorption maximum at 280 nm, while, in the case of WPI-OE and WPI-Qu proteins, in addition to this peak, new shoulders in the UV–vis spectra at the maximum of absorption ~350 were also shown (Figure 3).

### 3.2. Characterization of the Change in the Chemical Properties of Modified Proteins

**Figure 4:**
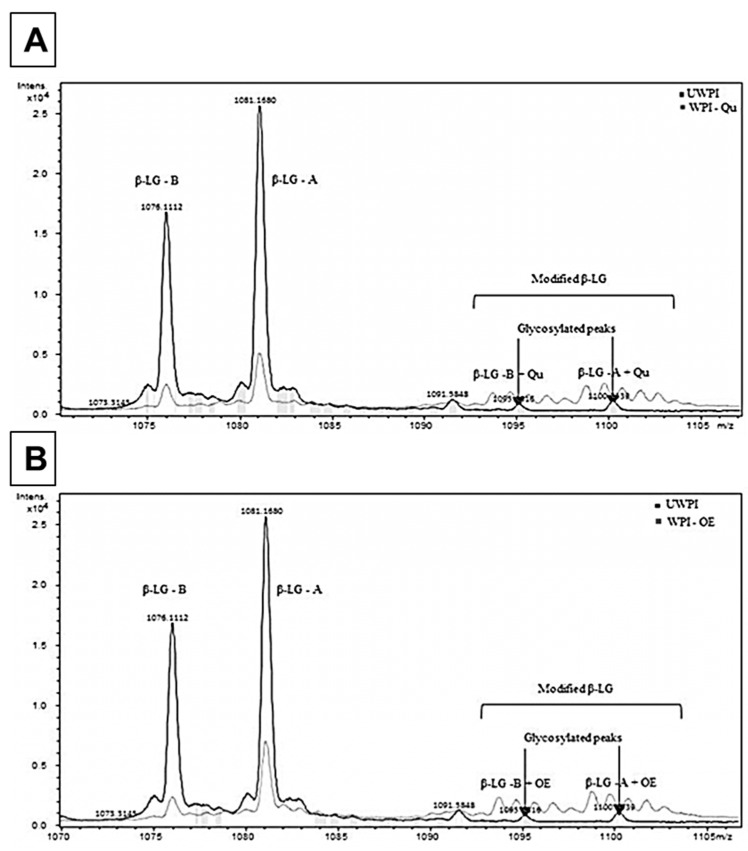
ESI-MS of whey protein isolates modified with quercetin dihydrate (**A**) and onion extract (**B**). UWPI, unmodified protein; WPI-Qu, modified WPI with quercetin; WPI-OE, modified WPI with onion extract.

Using the LC-ESI-TOF-MS method, the changes in molecular weight of WPI protein after 24 h of incubation with OE and Qu at pH 9 were investigated (Figure 4A,B). According to signal intensities, the signals at charge state + 17 at *m/z* 1076.1112 (variant B) and *m/z* 1081.1680 (variant A) in the ESI-TOF mass spectra of unmodified and modified WPI indicate the existence of two major -LG protein variants present in nearly all equimolar ratios. Deconvolution of the signals leads to an average protein mass of 18,276.99 Da for variant B, and for variant A, an average mass of 18,362.45 Da was calculated. Furthermore, two minor peaks with masses of 18,602 and 18,687 Da were identified, which are linked to minor glycosylation of two variants of β-LG. After the modification of WPI with OE and Qu, new peaks were detected (Figure 4A,B), which account for the addition of one molecule of the Qu (*m/z* 301 Da) isomer to both β-LG protein variants. Moreover, the figures showed a significant decrease in the intensity of the two modified WPI compared to WPI.

### 3.3. Systemic Resistance Induced by Modified Proteins against Pepper mild mottle virus (PMMoV)

#### 3.3.1. Effects of Modified Proteins on the Severity and Concentration of PMMoV

The main criteria utilized to assess the infection with PMMoV disease were plant symptoms and virus accumulation. The incidence and severity of the PMMoV disease were evaluated and the quantity of the accumulated virus at 2 and 3 WAI was tested. Pepper plants infected with PMMoV exhibited severe mosaic and deformation in control after 3 WAI. Disease incidence and severity and ELISA results revealed that UWPI, WPI-QU and WPI-OE treatments significantly reduced disease severity and virus accumulation compared to the control (Table 2 and Table 3). Disease severity and ELISA values were substantially lower in WPI-QU than WPI-OE and UWPI. Additionally, ELISA data indicated that the treatments with WPI-OE resulted in a significantly lower concentration of PMMoV compared with UWPI treatment. The results exhibited that WPI-QU and WPI-OE treatments could positively affect PMMoV accumulation leading to disease reduction.

#### 3.3.2. Effect of Modified Whey Protein Treatments on the Expression of Defense-Related Genes

To evaluate whether UWPI, WPI-QU and WPI-OE activate systemic resistance and enhance defense response in pepper plants against PMMoV, we tested the expression levels of *PR4*, *PR9* (SA pathway), *PIN2* (jasmonic acid pathway) and *Tin1* (ethylene pathway).

**Figure 5:**
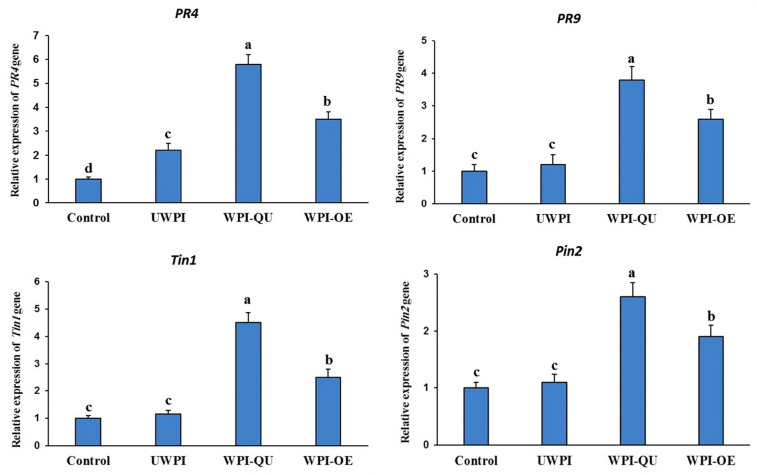
Expression of defence-related genes *PR4*, *PR9*, *Tin1* and *PIN2* in pepper evaluated by qRT-PCR at 1 day after modified protein treatment (just before PMMoV inoculation). *Actin* (the housekeeping gene) was used as a control. Different letters denote significant differences.

**Figure 6:**
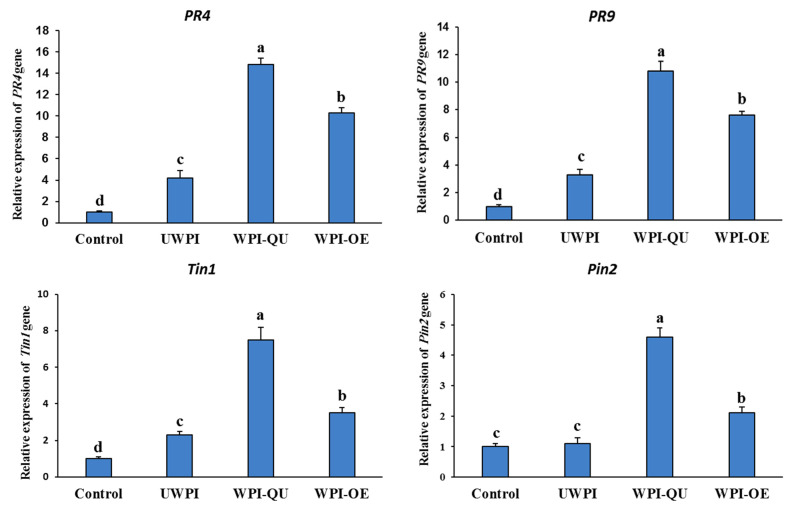
Expression of defence-related genes *PR4*, *PR9*, *Tin1* and *PIN2* in pepper evaluated by qRT-PCR at 1 day after PMMoV inoculation. *Actin* (the housekeeping gene) was used as a control. Different letters denote significant differences.

The expression levels of *PR4* gene revealed a 6- and 15-fold increase in plants treated with WPI-QU at 0- and 1-days post-inoculation (dpi), respectively, while 10- and 4-fold increases were found in WPI-OE and UWPI treated plants, respectively, at 1 dpi (Figure 5 and Figure 6). The expression levels of *PR9* were up-regulated 4- and 11-fold in WPI-QU treatment at 0 and 1 dpi, respectively. WPI-OE and UWPI up-regulated the expression of *PR9* to 8- and 3-fold, respectively, at 1 dpi (Figure 6). The expression levels of *PIN2* (JA signaling) were up-regulated 2.6- and 4.6-fold in WPI-QU treatment at 0 and 1 dpi, respectively. WPI-OE and UWPI up-regulated the expression of *PIN2* to 2- and 1-fold, respectively, at 1 dpi (Figure 6). The expression levels of *TIN1* (ET signaling) were up-regulated 5- and 7-fold in WPI-QU treatment at 0 and 1 dpi, respectively. UWPI and WPI-OE up-regulated the expression of *TIN1* to 2.3- and 3.5-fold, respectively, at 1 dpi (Figure 6). No significant difference was found between the control and UWPI treatments for *PIN2* expression levels at 1 dpi.

#### 3.3.3. Effect of Modified Whey Proteins on Total Phenol Contents and Defense Enzyme Activities

The effects of WPI-QU and WPI-OE on the content of total phenols and enzymes are presented in Table 4. Total phenols and defense enzymes were significantly increased in WPI-QU and WPI-OE treatments relative to the control. The maximum increase in total phenols was reported in plants treated with WPI-QU. Similarly, WPI-QU treatment offered the highest total phenol among the tested treatments. These results explained the important role of total phenol and defense enzymes in disease resistance.

#### 3.3.4. Effect of Modified Whey Proteins on Certain Plant Growth Parameters

UWPI, WPI-QU and WPI-OE were tested for their impact on pepper growth. When compared to the control group, plant development was substantially improved using UWPI, WPI-QU and WPI-OE treatments (Table 5). Pepper treated with UWPI, WPI-QU and WPI-OE were significantly taller than untreated control plants. The WPI-QU treatment enhanced the fresh and dry weights of the shoots to 9.35 g and 3.96 g, respectively, compared with the control (6.84 g and 2.37 g, respectively). Therefore, WPI-QU treatment outperformed WPI-OE and UWPI treatments in terms of increasing pepper growth.

#### 3.3.5. Effects of Modified Whey Proteins on Antioxidative Parameter and Vitamin C Contents

The total antioxidant status and vitamin C contents in pepper plants treated with UWPI, WPI-QU and WPI-OE, as well as the control, after the infection with PMMoV are displayed in Table 6. The TAS and vitamin C contents of the WPI-QU-treated plants were higher than those of WPI-OE and UWPI. Moreover, UWPI treatment resulted in lower values than WPI-OE. In general, all treatments exhibited improved values in TAS and vitamin C compared with the control.

## 4. Discussion

Several studies have suggested milk as a way to decrease the transmission of viral particles between plants [22]. Changes in the charges of milk proteins may improve their antiviral capabilities due to chemical alterations [23]. Fluorescence quenching spectroscopy is a valuable method for analyzing the covalent interactions between phenolic compounds and proteins. Determining the change in fluorescence emission intensity gives information about the changes in the structure and conformation of protein [24,25]. The unmodified WPI had the highest fluorescence intensity at 356 nm. The maximum emission wavelength of modified WPI proteins, on the other hand, changed very little, with readings of 363 and 365 nm for WPI modified with onion extract (OE) and quercetin (Qu), respectively. These results showed that the interaction between Qu and WPI proteins was higher than OE. Oxidized phenolic chemicals and tryptophan residue may interact [25,26]. Moreover, the WPI-Qu conjugate showed the highest intensity values compared to UWPI and WPI-OE. Since unbound Qu was removed through the extensive dialysis process, it could be concluded that these new shoulders are correlated to the covalently bound OE and Qu to WPI protein. This is the first study to discover the products of covalent interactions between OE and WPI proteins under alkaline (pH9) conditions, as far as we know. For variation B, deconvolution yielded an average protein mass of 18,276.99 Da, whereas, for variant A, an average mass of 18,362.45 Da was calculated. These findings are consistent with what others have reported [27,28]. It was impossible to tell which amino acids had been changed with Qu based on the mass spectra of intact proteins. The modified proteins were submitted to tryptic digestion followed by MALDI-MS or ESI-MS to identify Qu binding sites [27,28]. They used tryptic digestion on coffee and Beta-lactoglobulin that had been treated with chlorogenic acid and allyl isothiocyanate, respectively.

In comparison to the control, the treatments of UWPI, WPI-QU and WPI-OE substantially decreased disease severity and viral accumulation. The obtained results disclosed the additive effect of modified whey proteins with quercetin and onion extract on the reduction of PMMoV severity and accumulation in pepper plants. Similarly, milk components and whey proteins were successfully used to control foliar diseases [22,29]. Cucumber mosaic virus (CMV) infection was controlled by reducing of disease severity and virus accumulation in leaves [13]. Additionally, whey treatments was efficient in controlling powdery mildew in cucumber and zucchini [30]. Conidia were ruptured by lactoferrin (a milk antibacterial component), but hyphae were not damaged until 48 h following treatment. The findings validate the theory that lactoferrin activity and free radical production correlate to milk’s ability to prevent powdery mildew [31].

Although the antimicrobial effect of whey protein is well documented, the mechanism of action against plant viruses remains unclear. PR genes are key players in plant defense and are often used to explain the mechanism of induced resistance. In plants treated with modified whey proteins, the expression levels of pathogenesis-related genes *PR4*, *PR9* (SA responsive genes), *PIN2* (jasmonic acid responsive gene), and *Tin1* (ethylene responsive gene) were all significantly elevated. *Trichoderma asperellum* SKT-1 enhanced the resistance against CMV in *Arabidopsis* through the activation of pathogenesis-related genes [13]. PR proteins are important for the synthesis of various secondary metabolites, and the accumulation of these secondary compounds is included in activated plant immunity and induced resistance [32].

Among antioxidant components in different crops, phenol content and antioxidant enzymes are the most important [33]. In comparison to the control, total phenols and defense enzymes were substantially higher in the WPI-QU and WPI-OE treatments. Defense enzymes are a physiological index of the stimulated resistance in different plants [34]. POD and PPO activities of WPI-QU and WPI-OE treated peppers displayed increasing levels after PMMoV inoculation. POD and PPO activities in WPI-QU treated plants revealed higher values than WPI-OE and control treatments. However, high levels of total phenol, POD and PPO may promote pepper resistance to various diseases [35].

WPI-QU treated plants had greater TAS and vitamin C levels than WPI-OE and UWPI treated plants. Most vegetables contain high levels of antioxidant capacity [36]. Pepper is a valuable crop because it is an important supply of vitamins and antioxidants [37]. Nevertheless, plants respond to biotic stress such as diseases through several defense mechanisms for detoxifying O^−2^ and H_2_O_2_ [38]. Many reports revealed the effect of virus infection on plant antioxidant capacity and growth conditions [39].

## 5. Conclusions

The structure of modified proteins was changed by covalent modification of whey protein isolate with quercetin and onion extract in alkaline conditions (pH 9), at room temperature in the presence of air. In comparison to OE, Qu exhibited a strong response as measured by RP-HPLC and ESI-MS. Polyphenol-enriched extracts may be produced from a variety of agro-industrial by-products, making this application especially attractive. These protein conjugates’ multifunctional properties make them suitable inducers for systemic resistance against PMMoV. This information may be interesting enough to motivate the current work. PMMoV infection in pepper may be controlled by developing systemic resistance, which reduces the disease severity and PMMoV accumulation in the treated plants. Pepper growth was enhanced by the application of modified whey proteins. It is possible to use WPI-QU and WPI-OE to suppress PMMoV in pepper plants.

## Figures and Tables

**Table 1:** Forward and reverse primers sequence for pathogenesis-related genes.

Gene	Forward Primer	Reverse Primer
*Tin1*	AGCCTGAAATAGAAGAAACGGAGATGGAGATGAG	GGAACCAGAATTGGTTACTCATGGCTACCTGAAC
*PIN2*	TGGGACTTTCATTTGTGAAGGAGAG	GACACAGTGAATAGGCAATATTTGG
*PR4*	AACTGGGATTGAGAACTGCCAGC	ATCCAAGGTACATATAGAGCTTCC
*PR9*	GACTAGTTTCAAGAGCATCA	AATTGTATAGCCTGTAGCTG
*Actin*	CACTGAAGCACCCTTGAACCC	GAGACAACACCGCCTGAATAGC

**Table 2:** Disease incidence (DI) and severity (DS) of PMMoV on pepper plants at 2 and 3 weeks post-virus inoculation (WPI).

Treatment	2 WPI		3WPI	
	DI (%)	DS	DI (%)	DS
UWPI	25b	2.4b	40b	2.9b
WPI-QU	12d	1.1d	20d	1.4d
WPI-OE	18c	1.8c	28c	2.1c
Control	68a	4.3a	89a	5a

Different letters denote significant differences.

**Table 3:** PMMoV accumulation in young leaves of pepper plants at 2 and 3 weeks after virus inoculation.

Treatment	2 WPI	3WPI
UWPI	0.54b	0.81b
WPI-QU	0.21d	0.47d
WPI-OE	0.39c	0.60c
Control	1.04a	1.49a

Different letters denote significant differences.

**Table 4:** The activities of peroxidase and polyphenol oxidase and total phenol contents in pepper plants elicited with WPI-QU or WPI-OE.

Treatment	Phenol (mg g^−1^ FW)	POX (U g^−1^ FW)	PPO (U g^−1^ FW)
UWPI	0.509c	0.562c	0.282c
WPI-QU	0.738a	0.896a	0.401a
WPI-OE	0.643b	0.782b	0.312b
Control	0.311d	0.414d	0.131d

Different letters denote significant differences.

**Table 5:** Effects of WPI, WPI-QU or WPI-OE on some plant growth parameters of pepper plants under greenhouse conditions.

Treatment	Plant Height (cm)	No. Leaves/Plant	Shoot Weight (g)	
			Fresh	Dry
**Control**	19.52d	9.03c	6.84d	2.37d
UWPI	24.26c	13.46b	8.14c	2.98c
WPI-QU	31.00a	15.32a	9.35a	3.96a
WPI-OE	28.16b	14.93a	8.61b	3.45b

Different letters denote significant differences.

**Table 6:** Comparison between the oxidative–antioxidative parameters and vitamin c contents of peppers.

Treatment	TAS, µmol Trolox Eq g ^−1^ Fwt	Vitamin C, µmol g ^−1^ Fwt
UWPI	4.6c	3.1c
WPI-QU	6.8a	4.2a
WPI-OE	5.5b	3.9b
Control	3.5d	2.4d

Different letters denote significant differences.

## Data Availability

Not applicable.
